# *Alu* expression in human cell lines and their retrotranspositional potential

**DOI:** 10.1186/1759-8753-3-11

**Published:** 2012-06-20

**Authors:** Andrew J Oler, Stephen Traina-Dorge, Rebecca S Derbes, Donatella Canella, Brad R Cairns, Astrid M Roy-Engel

**Affiliations:** 1 Tulane Cancer Center SL-66, Dept. of Epidemiology, Tulane University, 1430 Tulane Ave, New Orleans, LA 70112, USA; 2 Department of Oncological Sciences, Huntsman Cancer Institute, and Howard Hughes Medical Institute, University of Utah School of Medicine, Salt Lake City, UT USA; 3 Center for Integrative Genomics (CIG), Faculty of Biology and Medicine, University of Lausanne, Lausanne 1015, Switzerland; 4 Bioinformatics and Computational Biosciences Branch, Office of Cyber Infrastructure and Computational Biology, National Institute of Allergy and Infectious Diseases, National Institutes of Health, Bethesda, MD 20892, USA

**Keywords:** Alu source elements, Alu expression, RT-PCR, ChIP-seq, Retrotransposition, SINE

## Abstract

****Background**:**

The vast majority of the 1.1 million Alu elements are retrotranspositionally inactive, where only a few loci referred to as ‘source elements’ can generate new Alu insertions. The first step in identifying the active Alu sources is to determine the loci transcribed by RNA polymerase III (pol III). Previous genome-wide analyses from normal and transformed cell lines identified multiple Alu loci occupied by pol III factors, making them candidate source elements.

****Findings**:**

Analysis of the data from these genome-wide studies determined that the majority of pol III-bound Alus belonged to the older subfamilies Alu S and Alu J, which varied between cell lines from 62.5% to 98.7% of the identified loci. The pol III-bound Alus were further scored for estimated retrotransposition potential (ERP) based on the absence or presence of selected sequence features associated with Alu retrotransposition capability. Our analyses indicate that most of the pol III-bound Alu loci candidates identified lack the sequence characteristics important for retrotransposition.

****Conclusions**:**

These data suggest that Alu expression likely varies by cell type, growth conditions and transformation state. This variation could extend to where the same cell lines in different laboratories present different Alu expression patterns. The vast majority of Alu loci potentially transcribed by RNA pol III lack important sequence features for retrotransposition and the majority of potentially active Alu loci in the genome (scored high ERP) belong to young Alu subfamilies. Our observations suggest that in an *in vivo* scenario, the contribution of Alu activity on somatic genetic damage may significantly vary between individuals and tissues.

## Findings

*Alu* elements are major contributors to genomic instability [1] and genetic disease [2] due to their ability to generate new copies that randomly insert throughout the genome and to induce non-homologous recombination between different copies. When comparing copy numbers, Alu has been vastly more successful than other non-autonomous elements, such as the retropseudogenes and even the autonomous L1 element [3]. Alu-induced mutagenesis is responsible for the majority of the documented instances of human retroelement insertion-induced disease [2] and presents a retrotransposition rate estimated up to ten-fold higher than L1 [4,5]. The human genome contains over one million *Alu* inserts [3], which can be divided into subfamilies based on specific diagnostic nucleotides and their evolutionary period of activity [6,7]. About 80% of *Alu* elements belong to the older previously active *Alu* J and *Alu* S subfamilies [6]. Germline derived evidence supports the current activity of only the subsets of the younger *Alu* Y subfamilies (such asY, Ya, and Yb)[8], although recent data appear to indicate that Alu retrotransposition in germline and somatic tissues may show different distributions [9].

**Figure 1:**
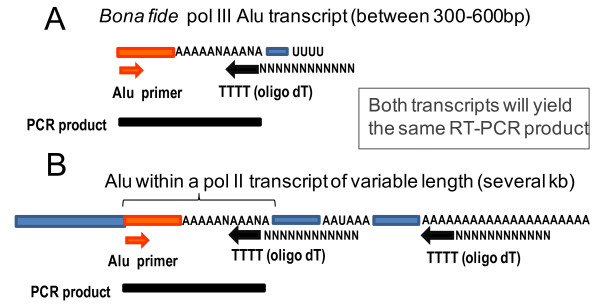
**RT-PCR assays are unable to distinguish between RNA pol III transcribed Alu transcripts and RNA pol II transcribed mRNAs containing Alu sequences**. **A.** Schematic of a *bona fide* pol III Alu transcript. Transcripts of 300 to 600 bp in length contain an Alu body (orange) flanked at the 3′ end by a poly-A stretch and the unique region (blue) which is determined by the location of the pol III terminator (Us). The poly-A stretch may be either homogeneous or heterogeneous containing non-adenosine bases. **B.** Schematic of an mRNA containing an Alu sequence. The Alu (orange) present within the mRNA (blue) will also have an oligo dA stretch at its 3′ end. Most standard RT-PCR approaches, such as 3′ RACE, rely on generating a cDNA through the reverse transcription of the RNA using an oligo dT primer (black arrow). Because both types of transcripts (pol II versus pol III) contain Alu sequences flanked by a polyA stretch, both will be amplified during reverse transcription. PCR amplification of selected cDNAs can then be performed by using a gene specific primer (in this case Alu, shown as an orange arrow) and a primer to the 3′ sequence of the oligo dT (represented as ‘Ns’). The PCR products (shown as black bars) of the cDNAs generated by both types of transcripts will yield the same type of product, thus making it difficult to distinguish the data generated from the *bona fide* pol III Alu transcripts.

Only a few Alu elements, referred to as ‘source’ or ‘master’ elements, undergo retrotransposition. Identification of source Alu elements has been elusive, as *bona fide* Alu retrotransposition events never present 5′ or 3′ transductions that could help determine a parent locus. Because transcription by RNA polymerase III (pol III) is necessary for Alu retrotransposition, a first step to identify a source element is to determine the transcribing Alu loci. There are little available data on RNA pol III transcribed Alu loci. Current techniques using RT-PCR approaches are unable to distinguish *bona fide* pol III Alu transcripts from those pol II transcripts containing Alu sequences (see Figure 1 for details). One of the few sources of reliable information was generated using primer extension and C-tail RACE, which showed a limited amount of SINE expression *ex vivo* in some cell lines [10,11]. Recently, genome-wide chromatin immunoprecipitation (ChIP) analyses followed by parallel sequencing (ChIP-seq) performed by three different laboratories identified multiple Alu loci bound by RNA polymerase III factors [12-14]. These datasets (Table 1) were generated from a variety of cell lines including a relatively ‘normal’ cell line: IMR90 (a Tert-immortalized, untransformed human lung fibroblast) and the tumor-derived cell lines: HeLa (cervical adenocarcinoma), Jurkat (T-cell leukemia) and K562 (myelogenous leukemia). Although the binding by pol III factors is not synonymous with transcription, these Alu loci represent potential candidate source elements.

**Table 1:** Data Source of Alu loci

**Study**	**Method**	**Cell lines**
Canella et al. [13]	ChIP-seq^a^ for detection of sites bound by POLR3D	IMR90
	(RPC4), TFIIIB subunits BDP1 and BRF1	
Oler et al. [12]	ChIP-seq^a^ and ChIP-array^b^ for detection of sites bound by Pol III (RPC32 subunit), TFIIIC63 subunit, BRF1, BRF2	HeLa, Jurkat, HEK, 293 T
Moqtaderi et al. [14]		
	ChIP-seq^a^ for detection of sites bound by TFIIIC-110	HeLa, K562
	subunit, TFIIIB subunits BDP1 and BRF1, Pol III (RPC155 subunit) and BRF2	

^a^Chromatin immunoprecipitation followed by massively parallel sequencing; ^b^chromatin immunoprecipitation followed by complementary DNA microarray hybridization. ChIP, chromatin immunoprecipitation.

**Figure 2:**
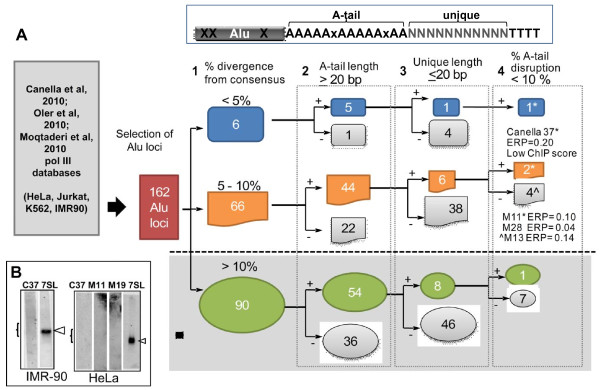
**Classification strategy of potentially active Alu loci.** A schematic of an Alu element is on the top right, where the body is shown in gray. X represent sequences changes differing from the consensus or non-A residues in the A-tail. Ns represent the unique flanking sequence located downstream of the A-tail and the first RNA polymerase III terminator defined as four or more thymidine residues (TTTT). **A**. Flow chart used for the identification of potentially retrotransposition competent Alu elements. Datasets from previously published data sets were interrogated for Alu loci identified as bound by RNA polymerase III factors. A total of 162 Alu-candidate loci were retrieved and classified based on four parameters: **1**- % divergence from the consensus sequence, which was subdivided into three categories based on the % sequence divergence from their Alu subfamily consensus (less than 5% in blue, 5 to 10% in orange and over 10% in green also classified as retrotranspositionally inactive). **2-** A-tail length, where Alu elements with A-tail lengths of equal to or over 20 bp were included as potentially active (+). Alu elements not meeting the criteria were binned separately and not further evaluated (shown as gray symbols; -). **3-** Length of the unique sequence, scoring positive Alu elements with 20 bp or less. **4-** % disruption of the A-tail sequence was scored selecting for those with 10% or less disruption. The two Alu candidates evaluated for expression (show in B) are indicated by the asterisks in the flow diagram. ERP = estimated retrotranspositional potential. **B**. Expression of candidate Alu loci with retrotransposition potential. Total RNA extracts from HeLa or IMR90 cells were hybridized with radioactively endlabeled oligomers complementary to the unique region of the Alu loci (Additional file 1: Table S11) following a previously published protocol [24]. Results from Alu Yb8 Canella 37 (C37), Alu Sx Moq 11 h (M11), and Alu Sx Moq 19 k (M19) are shown. An oligo complementary to a region of the 7SL RNA that does not share sequence similarity to Alu RNA: 5′-CCGATCGGCATAGCGCACTA-3′ was used as a positive control (white arrowhead). Bracket approximates the expected location of the Alu transcript.

To evaluate these candidate elements, we retrieved the Alu-related sequences for those enriched with pol III initiation factors (pol III or TFIIIB) in the published datasets [12-14] including the ‘A-tail’ and ‘unique’ region at the 3′ flanking sequence (see schematic of an Alu in Figure 2). Each pol III bound Alu locus was assigned a name based on the dataset and/or cell line where it was identified. The 3′ flanking sequence included either 300 bp or up to the first pol III terminator (which was defined as four or more thymidine residues) of the downstream genomic flanking sequence (complete data set shown in Additional file 1: Tables S4-S9). We then selected only those that fit the standard dimeric Alu structure, eliminating any FLAMs, FRAMs and partial Alu elements. In addition, we eliminated any Alu sequences that contained an internal pol III terminator as these would generate truncated Alu transcripts.

A total of 162 Alu elements fit our criteria (Additional file 1: Table S1). Several loci (24 out of 162, 14.8%) were identified in at least two separate cell lines (Additional file 1: Table S2), suggesting potential regions preferentially bound by RNA polymerase III factors. Each Alu locus is represented only once in our data set and analyses. Although the majority of Alu elements in the genome belong to the older Alu subfamilies (S + J) currently, only the ‘young’ Alu subfamilies appear to be retrotranspositionally active. Classification of the dataset of pol III-bound Alus revealed that the majority belong to the older subfamilies (Table 2) consistent with previously published expression data [11]. Although the Alu subfamily distribution from each individual cell line showed variation, the old Alu S + J subfamilies represented at least two thirds and up to 98% of the identified loci (Additional file 1: Table S3). When all Alus are considered together, a moderately significant association is found between pol III-binding and Alu J + S elements in at least one cell line but not Alu Y elements (odds ratio = 1.6, *P* = 0.098 in Fisher’s exact test), suggesting that pol III is approximately 1.6 times less likely to bind to an Alu Y element than to an Alu J or S element. Normalization of the collective dataset for Alu subfamily copy number differences (older Alus are vastly more abundant than younger elements), we observe that proportionally, there are more young Alu elements bound by pol III factors (Table 2); however, these differences are not significant (*P* = 0.21 and *P* = 0.44 for AluYa5 and AluYb8, respectively, in Fisher’s exact test).

**Table 2:** Alu subfamily distribution of Alu elements bound by RNA polymerase III factors

**Alu**	**% Total**	**# of disease**	**% Alu**	**% Alu loci**	**Pol III**	**# Pol III**
**subfamily**^**a**^	**full Alus (530,850)**	**cases due to a*de novo insertion***	**transcripts**^**d**^	**bound by pol III**	**bound Alu enrichment**	**bound Alu with ERP**
		**(%)**^**c**^		**factors**^**e**^		≥0.10
**S + J**	84.5	0 (0)	66	88.4	1.0	2
**Y**	15.5	13 (23)	33	9.8	0.6	0
**Ya5**	0.63	24 (44)	0.8	1.2	2.0	0
**Yb8**	0.42	18 (33)	0.5	0.6	1.5	1

^a^Includes all subfamily variants: for example, AluYa5, AluYa5a2, AluYa8 are classified as Ya5; ^b^number of Alu elements meeting full length criteria (Details in supplemental data); ^c^subfamily distribution of the 55 *de novo* Alu elements reported to cause a disease [2,15]; ^d^analysis was performed on transcripts isolated from the Ntera2 (teratocarcinoma) cell line [11]; ^e^analyses were performed on the cumulative data obtained from studies on IMR-90 (normal untransformed Tert-immortalized lung fibroblast) [13], HeLa (cervical adenocarcinoma), Jurkat (T-cell leukemia), HEK 293 T (T antigen-transformed kidney) [12] and K562 (myelogenous leukemia) cells [14] (raw data and detailed analysis for each cell line included in Additional file 1: Table S3); ^f^represents the increase in Alu loci bound by RNA polymerase III factors relative to copy number (that is, %AluY bound/%AluY copies in the genome, detailed analysis in Additional file 1: Table S3); ^g^number of Alu loci bound by RNA polymerase III factors with estimated retrotransposition potential (ERP) scores of ≥0.10. An ‘ideal’ Alu will have ERP score of 1.00.

In addition to the ability to be transcribed, specific sequence features of Alu elements can influence retrotransposition efficiency [22]. Therefore, we proceeded to evaluate the individual pol III-bound Alu loci using our own designed dichotomous key based on the previously identified criteria known to affect retrotransposition rates: 1) sequence divergence from the consensus (loss of retrotransposition efficiency with higher divergence [22,16]); 2) A-tail length (a minimum length is required [17]); 3) length of the unique sequence (loss of efficiency with longer sequences [22]); and 4) A-tail homogeneity (loss of efficiency with higher % disruptions [22]). Our results are schematically represented in Figure 2A (details in Additional file 1: Table S1). We selected limits for our criteria parameters that have been shown to significantly reduce retrotransposition levels. We also separately assigned a numerical value of the impact on retrotransposition (‘R’) for each Alu feature variant relative to an Alu reference (Additional file 1: Tables S12-15) to roughly calculate the ERP of each individual Alu (Additional file 1: Table S1, column T). However, the ERP should not be taken as the sole defining criteria for *in vivo* predictions, as it is based on a limited amount of data generated from engineered Alus in a tissue culture system and does not include transcription status. This scoring system was applied to the pol III-bound subset as well as all Alus genome-wide using an algorithm that incorporates each of the scoring criteria (implemented in Perl, score_alus.pl; available upon request). As expected, young Alu elements had a higher score genome-wide than Alu J + S elements (median values of 0.0042 and 0.000001 for Y and J + S, respectively; *P* = 2.2e-16 in Wilcoxon test). While pol III-bound Alus had a higher ERP score in general than Alus not bound by pol III (median 0.000229 and 0.000001, respectively; *P* = 0.013 in Wilcoxon test), the ERP score for the vast majority of the pol III-bound Alus was considerably lower than the arbitrarily selected minimal threshold for retrotransposition competency of 0.20. Of the 162 pol III-bound Alu sequences only one AluYb8 (Canella 37 from IMR90 cells) was highly conserved relative to the consensus sequence, met the rest of the criteria and scored 0.20 ERP (an ‘ideal’ Alu will have a score of 1.00). In addition, it scored low in the pol III ChIP assay [13] and Canella 37 AluYb8 transcripts were undetectable in HeLa and IMR90 cells by northern blot probing with end-labeled oligonucleotides complementary to the unique sequence (Figure 2B). We opted not to use an RT-PCR approach, as it is unable to differentiate between RNA pol II and pol III transcripts (Figure 1). In contrast, when using a low ERP threshold to evaluate the reference genome, several thousands of Alus genome-wide were identified (6,103 and 1,818 Alus at ERP threshold of 0.10 or 0.20, respectively; Additional file 1: Table S16). Furthermore, a more conservative threshold (ERP scores of ≥0.50) yields only 163 of genome-wide Alus (all young elements), corroborating the previously proposed Alu source model that only a small portion of Alus in the genome are likely active [18].

The next ‘best’ candidates identified only partially met the criteria, corresponding to three Alu loci belonging to older S subfamilies: Moq 13 h (HeLa), Moq 11 h (HeLa) and Moq 28 k (K562) with 5.3%, 7.8% and 9.3% sequence divergence from consensus, respectively. Some of the sequence changes were within the RNA pol III A box and in the sequences predicted to bind the SRP9 and SRP14 proteins. Lower binding of SRP9/14 would likely reduce the retrotransposition capability of these elements, but further testing is required. Moq 28 k shows a very low ERP of 0.04. Interestingly, Moq 11 h and Moq 13 h present acceptable A-tail length with marginal ERP values of 0.10 and 0.14, respectively. Moq 13 h showed an A-tail with high % A-tail disruption (24.3%), which is not observed in *de novo* inserts [19]. The published work on Moq 11 h showed significant pol III binding by ChIP-seq [14]. If expressed, Moq 11 h could prove retrotranspositionally competent. However, the RNA-seq data showed only three sequence reads in HeLa and none in the K562 and a non-detectable transcript by northern blot analysis (Figure 2B). Evaluation of expression from five other randomly selected Alu loci, Moq 19 k (Figure 2B) and Canella 2 and 28, Oler 38 h, and 3c, (data not shown) by northern blot analysis also proved unsuccessful in the detection of pol III Alu transcripts. Due to the sensitivity limitation of our assay, we are unable to unambiguously confirm that these identified Alu candidate loci with the best retrotransposition potential (Canella 37 and Moq 11 h) are transcriptionally silent. Thus, we cannot eliminate the possibility that very low amounts of expression may occur, resulting in retrotransposition. Alternatively, these or other identified Alu loci may be more efficiently expressed in other cell types, tissues or under other conditions such as heat shock known to increase Alu expression [20].

Presently, we are unable to rule out that any of the other identified pol III-bound Alu candidates that partially fulfill our criteria or contain borderline attributes may undergo retrotransposition at very low rates. However, the limits of the criteria are based on the results using a tissue culture system [21] that significantly favors Alu activity through the overexpression of both a tagged Alu transcript and the enzymatic machinery required for retrotransposition(L1 ORF2 protein). This opens the possibility that an Alu locus identified as potentially active by the selected parameters may not be able to retrotranspose under natural cellular conditions. Thus, it is unlikely that the ‘less perfect’ Alu candidate elements (those with low ERP scores) contribute to retrotransposition in any significant manner.

Our findings indicate that up to now, most cells analyzed may support RNA pol III expression of a collection of Alu elements, although the vast majority lack sequence features associated with retrotransposition competence (Table 2). A striking observation is the overall low number of detected Alu loci (162), and even lower when considering retrotransposition potential (only three loci from all cell lines combined had ERPs above 0.10). So why is there little to no evidence of expression by pol III of the active younger Alu elements? Although speculative, these data could be indicative of a general mechanism, such as DNA methylation, that selectively limits Alu transcription of the retrotranspositionally competent elements. Also, it could be a reflection that younger, less mutated retroelements still maintain most of their CpGs making them good substrates for regulation by methylation [22]. In addition, the inability to detect transcripts from the candidates identified may reflect variability in Alu expression, where the same cell lines in different laboratories have different expression patterns. It is possible that Alu expression varies by cell type, growth conditions, epigenetic signals and transformation state. Our observations support the hypothesis that in an *in vivo* scenario, the contribution of Alu activity on somatic genetic damage may significantly vary between individuals and tissues.

## Competing interests

The authors declare that they have no competing financial interests.

## Authors’ contributions

AMR-E designed the experimental approach, directed, and performed analyses. ST-D mined some of the Alu sequences and performed the northern blot analyses, with the assistance of RSD. AJO mined the Alu sequences from the Moqtaderi data files, performed the reanalysis of ChIP-seq data, and wrote the algorithm for classification of Alus and ERP score. AJO and BRC provided RNA-seq data. AJO, BRC and DC provided scientific consultation on data interpretation and writing. All authors read and approved the final manuscript

## Supplementary Material

Additional file 1 **Table S1.** Final Alu data set. **Table S2.** Duplicate Alu loci (Alu locus was identified in more than one cell type). **Table S3.** Alu subfamily distribution. **Table S4.** Retrieved Alus from HeLa Table 1b Pol3 ChIP-chip in HeLa from Oler,A.J. et al. Nat Struct Mol Biol 17, 620–628 (2010). **Table S5.** Retrieved Alus from Table 1c HeLa ChIP-seq Knowns from Oler, A.J. et al. Nat Struct Mol Biol 17, 620–628 (2010). **Table S6.** Retrieved Alus from Table 1 g Pol3 Jurkat ChIP-seq from Oler, A.J. et al. Nat Struct Mol Biol 17, 620–628 (2010). **Table S7.** Retrieved Alus from Table 1 h Pol3 multiple cell types from Oler, A.J. et al. Nat Struct Mol Biol 17, 620–628 (2010). **Table S8.** Retrieved Alus from IMR90, Table S7 from Canella,D., et al. Genome Res. 20, 710–721 (2010). **Table S9.** Retrieved Alus from HeLa cells, Moqtaderi, Z, et al. Nat Struct Mol Biol, 20, 635–640 (2010). **Table S10.** Retrieved Alus from K562 cells, Moqtaderi, Z, et al. Nat Struct Mol Biol, 20, 635–640 (2010). **Table S11.** Probes for northern blot analysis. **Table S12.** Numerical value assigned base on the impact of Alu sequence divergence on retrotransposition potential. **Table S13.** Numerical value assigned base on the impact of A-tail length on retrotransposition potential. **Table S14.** Numerical value assigned base on the impact of length of unique sequence on retrotransposition potential. **Table S15.** Numerical value assigned base on the impact of A-tail disruptions on retrotransposition potential. **Table S16.** Output of genome-wide analysis of B box-containing Alus with ERP (Competence_score) > = 0.10. Click here for file
